# Myxobacteria: Moving, Killing, Feeding, and Surviving Together

**DOI:** 10.3389/fmicb.2016.00781

**Published:** 2016-05-26

**Authors:** José Muñoz-Dorado, Francisco J. Marcos-Torres, Elena García-Bravo, Aurelio Moraleda-Muñoz, Juana Pérez

**Affiliations:** Departamento de Microbiología, Facultad de Ciencias, Universidad de GranadaGranada, Spain

**Keywords:** *Myxococcus xanthus*, motility, predation, prokaryotic development, multicellularity

## Abstract

*Myxococcus xanthus*, like other myxobacteria, is a social bacterium that moves and feeds cooperatively in predatory groups. On surfaces, rod-shaped vegetative cells move in search of the prey in a coordinated manner, forming dynamic multicellular groups referred to as swarms. Within the swarms, cells interact with one another and use two separate locomotion systems. Adventurous motility, which drives the movement of individual cells, is associated with the secretion of slime that forms trails at the leading edge of the swarms. It has been proposed that cellular traffic along these trails contributes to *M. xanthus* social behavior via stigmergic regulation. However, most of the cells travel in groups by using social motility, which is cell contact-dependent and requires a large number of individuals. Exopolysaccharides and the retraction of type IV pili at alternate poles of the cells are the engines associated with social motility. When the swarms encounter prey, the population of *M. xanthus* lyses and takes up nutrients from nearby cells. This cooperative and highly density-dependent feeding behavior has the advantage that the pool of hydrolytic enzymes and other secondary metabolites secreted by the entire group is shared by the community to optimize the use of the degradation products. This multicellular behavior is especially observed in the absence of nutrients. In this condition, *M. xanthus* swarms have the ability to organize the gliding movements of 1000s of rods, synchronizing rippling waves of oscillating cells, to form macroscopic fruiting bodies, with three subpopulations of cells showing division of labor. A small fraction of cells either develop into resistant myxospores or remain as peripheral rods, while the majority of cells die, probably to provide nutrients to allow aggregation and spore differentiation. Sporulation within multicellular fruiting bodies has the benefit of enabling survival in hostile environments, and increases germination and growth rates when cells encounter favorable conditions. Herein, we review how these social bacteria cooperate and review the main cell–cell signaling systems used for communication to maintain multicellularity.

## Introduction

The existence of multicellular organisms in all the lineages of the tree of life suggests that multicellularity emerged on multiple occasions in the course of evolution (Rokas, 2008; Aravind et al., 2009). In the prokaryotic domains (*Bacteria* and *Archaea*), multiple emergences of multicellularity have also been observed (Grosberg and Strathmann, 2007). These prokaryotes,though simple in their architecture and morphology, and with only a small number of differentiated cells types, greatly resemble higher multicellular organisms. Although the cells in clonal populations of multicellular prokaryotes share the same genetic material, cells can differ significantly from one another in their properties and behaviors. The phenotypic variations between identical individuals are mainly due to the regulation of gene expression in response to different microenvironments, but they can also be a consequence of random cellular variability due to unavoidable stochastic fluctuations in genetic circuits that regulate cellular functions (Eldar and Elowitz, 2010; van Vliet and Ackermann, 2015). The most important benefit of such phenotypic variations is the division of labor, with different cell types specialized in different functions working together. Such division of labor, combined with cell–cell adhesion and coordinated intercellular communication, permits the whole population to function more efficiently, to achieve new synchronized functionalities, and to develop complex group behaviors, such as avoidance of predation and of non-cooperative individuals and improvement in efficiency of nutrient acquisition (Ackermann et al., 2008; Aguilar et al., 2015).

There are numerous unicellular microorganisms that display incipient multicellularity, such as the formation of filaments or simple clusters. These types of manifestations may be the result of simple aggregation that requires an extracellular matrix (ECM), incomplete cell fission after division, or formation of cells joined at their ends that share the periplasm or even the cytoplasm (Claessen et al., 2014; Lyons and Kolter, 2015). Examples are found in unicellular fungi such as *Saccharomyces cerevisiae*, archaea such as *Methanosarcina*, and many bacteria of the phyla *Cyanobacteria*, *Actinobacteria*, *Chloroflexi*, *Proteobacteria*, and *Firmicutes* (Macario and Conway de Macario, 2001; Claessen et al., 2014; Lyons and Kolter, 2015). Another class of multicellularity is the formation of more stable aggregates, which includes the formation of biofilms and swarms. This class is widespread among bacteria such as *Bacillus* and *Proteus* (Lyons and Kolter, 2015). Likewise, there is a smaller number of species that display even more complex multicellularity (such as *Caulobacter*, *Pseudomonas*, and myxobacteria), which consists of the construction of patterned multicellular structures. This complex behavior requires self-recognition, spatial morphogenesis, cell differentiation, division of labor, intercellular communication, and cooperation among individual cells (Kearns, 2007; Lopez and Kolter, 2010; Koschwanez et al., 2011; Claessen et al., 2014; Lyons and Kolter, 2015). These bacteria are well-organized cooperators that function more efficiently as multicellular units.

Myxobacteria are one of the bacterial groups that have effectively made the transition from single cell to multicellular life, exhibiting multifaceted cooperative behaviors and multicellular development comparable in sophistication to that seen in macroscopic social organisms. In depleted conditions, they form multicellular biofilms called fruiting bodies that vary from simple mounds to convoluted three dimensional structures, within which some bacteria altruistically develop into non-reproductive cells, while others differentiate into resistant and reproductive spores (Shimkets, 1999). Furthermore, their multicellular behavior encompasses other aspects of their life cycle such as mass predation and cooperative motility. Myxobacteria, particularly during the fruiting body formation process, represent an interesting case on the path to obligate multicellularity. Cooperation is not strictly necessary in a favorable environment, nor they do enter into a multicellular state without the appropriate conditions, such as high cell density, a solid surface, and starvation. Therefore, multicellularity in myxobacteria is transitory, and not obligatory, as opposed to obligate multicellularity, where organisms have no choice but to be multicellular (António and Schulze-Makuch, 2012). Indeed, Velicer et al. (1998) demonstrated by using laboratory experimental evolution that multicellularity in myxobacteria can be lost if it is not needed. Under conditions in which multicellularity is not advantageous (e.g., liquid, shaken cultures), defects in fruiting body formation, sporulation and motility only emerged after 1000 generations, suggesting that these social behaviors were all insignificant for fitness.

## *Myxococcus xanthus*, A Model for Multifaceted Cooperative Behaviors in Bacteria

**FIGURE 1:**
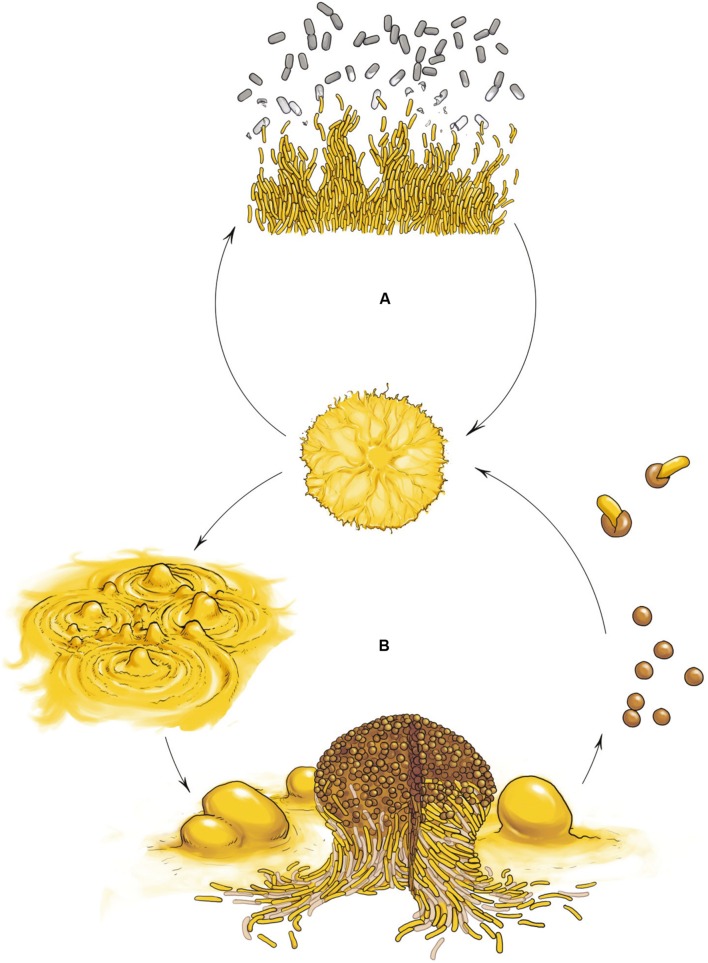
***Myxococcus xanthus* multicellular cell cycle.** **(A)** Vegetative growth. In the presence of nutrients cells move in a coordinated manner, forming swarms. When swarms make contact with the prey, cells penetrates the prey colony and lyse the cells. **(B)** Developmental cycle. Upon starvation, cells moving collectively initiate a developmental program and exchange extracellular signals as well as physical contact signals to first form aggregates and later build millimeter-long upright fruiting bodies filled with differentiated, reproductive and environmentally resistant cells called myxospores (rounds cells), surrounded by two other subpopulations showing division of labor: a monolayer of aligned non-reproductive peripheral rods (yellow rod cells) and cells that undergo altruistic obligatory autolysis by programmed cell death (light brown rod cells). Myxospores ensure survival during starvation or desiccation and can be dispersed to other environments and germinate when nutrient conditions ameliorate.

**FIGURE 2:**
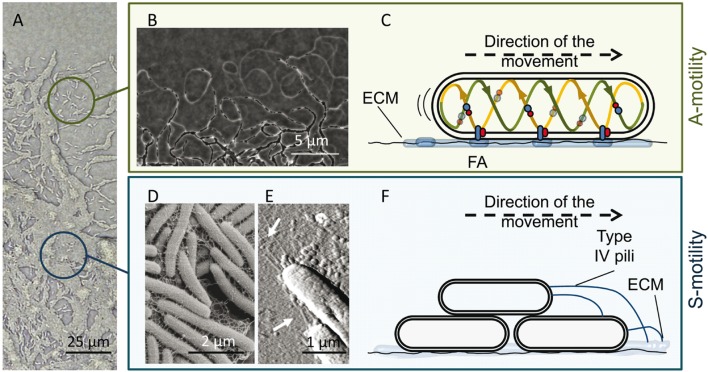
***M. xanthus* A and S motility.** **(A)** The edge of a *M. xanthus* swarm. Upper circle, single cells (with A-motility); bottom circle, group of cells (with S-motility). **(B)** Phase contrast microscopy revealing A-motility-mediated trails observed at the leading edge. Migration of other cells through these trails promotes the formation of dense regions of aligned cells and favors intimate cell–cell contacts. **(C)** Proposed focal adhesion (FA) model of gliding motility. The cytoplasmic, inner membrane and periplasmic components of the Agl–Glt motility protein complex move along a helical track (provided by cytoskeletal proteins) within the cells. After this trafficking, the complex engages the outer components and then the entire complex adheres to the substrate via ECM slime, forming an FA that allows the machinery to push. The protein complexes translocate along the cellular track, pushing the cell forward. **(D,E)** Components of the S-motility system, fibrils and type IV pili (T4P). **(D)** Scanning electron microscopy of the meshwork of fibrils that maintain cellular cohesion. **(E)** Atomic force microscopy of T4P localized at the leading cell pole. **(F)** Proposed model for S-motility. T4P anchors to the EPS present on neighboring cells and propels the cell by cycles of extension, attachment, and retraction. The pictures of the phase bright A-motility trails **(B)** and T4P **(E)** were adapted from Gloag et al. (2015) with permission of the authors, and from Pelling et al. (2005) with permission; copyright (2005) National Academy of Sciences, USA. Micrograph of the fibril material was kindly provided by L. J. Shimkets.

The best characterized myxobacterium is *Myxococcus xanthus.* Its life cycle comprises two phases that highlight the social nature of this organism: cooperative predation and multicellular development (**Figure 1**). Both multicellular processes are mediated by the coordinated movement of cells using two motility systems (**Figure 2**), individual motility (adventurous motility or A-motility) and group motility (social motility or S-motility), which are dealt with in the next section. In the presence of nutrients, cells move in a coordinated manner, forming multicellular biofilms known as swarms. When swarms make contact with prey, thousands of cells eventually penetrate the prey colony and lyse the cells (**Figure 1A**) (Berleman and Kirby, 2009; Pérez et al., 2016). This group predation strategy favors the swarm hydrolyzing extracellular biopolymers using common exoenzymes and, thus, making the most efficient possible use of the available sources of nutrition. However, upon starvation, cells moving collectively start a developmental process and exchange extracellular chemical signals as well as physical contact signals to form millimeter-long upright fruiting bodies (Kaiser, 2004; Mauriello et al., 2010). These mature multicellular structures (**Figure 1B**), filled with environmentally resistant myxospores (O’Connor and Zusman, 1991a), are surrounded by two different subpopulations showing division of labor (**Figure 1B**): a monolayer of aligned peripheral rods which are distinct from vegetative cells and spores (O’Connor and Zusman, 1991b), and cells that undergo altruistic obligatory autolysis through a developmentally programmed cell death (PCD; Wireman and Dworkin, 1977; Nariya and Inouye, 2008). Within the fruiting bodies the myxospores are firmly bound together, hence upon germination the whole population stays together to create a new community.

For both predation and development, this myxobacterium uses self-secreted ECM, intricate social networks, and cell–cell communication as the basis for its multicellular lifestyle. The ECM is a heterogeneous mix of secreted uncharacterized polysaccharides, a small protein fraction of unknown function, and extracellular DNA (Kearns et al., 2002; Bonner and Shimkets, 2006; Bonner et al., 2006; Curtis et al., 2007; Hu et al., 2012). ECM is required for sustaining *M. xanthus* multicellularity, not only to maintain the integrity of cell groups, which is important for biofilm formation, cellular cohesion and connection of cells to the substrate, but also because it participates in motility and fruiting body morphogenesis (Arnold and Shimkets, 1988a,b).

Over the entire life cycle, adjacent cells of *M. xanthus* are also interconnected by a network of outer membrane vesicles (OMVs) and outer membrane tubes (OMTs). Some authors consider that this array of lipid appendage-based bridges, which provides a flexible connection between cells, might play a functional role in cell-to-cell transfer of proteins through outer membrane exchange (OME) or in intercellular signaling (Palsdottir et al., 2009; Remis et al., 2014). The network of OMTs might provide an enlarged surface area for metabolite exchange, or even connect the periplasmic spaces of cells, although another possibility is that their occurrence in *M. xanthus* biofilms only plays a structural role by helping to physically bind cells together during social behaviors (Remis et al., 2014) or by acting as nucleation sites (Whitworth, 2011). Regarding OMVs, it has been proposed that free vesicles would provide a mechanism of signal transmission, while OMV chains could mediate direct intercellular contact creating a firmly bonded multicellular community. Purified OMVs contain lipids, fucose, mannose, *N*-acetylglucosamine, and *N*-acetylgalactosamine, and a small set of cargo proteins with hydrolytic activity and molecules associated with antibiotic activity (Berleman et al., 2014; Remis et al., 2014). The role of OMVs in helping mediate the killing of prey organisms through the delivery of these toxic proteins or antibiotics in cooperative predation is unquestionable (Berleman et al., 2014; Keane and Berleman, 2016), but they also contain proteins implicated in motility, such as CglB, and Tgl outer membrane proteins known to be transferable between cells (Hodgkin and Kaiser, 1979).

Outer membrane exchange is a novel myxobacterial mechanism that involves membrane fusion and the exchange of large amounts of outer membrane components among cells. By using OME *M. xanthus* cells share cell content to repair damaged siblings, leading to advantageous consequences for both the donor and the recipient (Vassallo and Wall, 2016). The potential role of OME in overcoming cell damage and as a social tool to make the transitions from unicellular free-living cells to multicellular populations has been reviewed by Wall (2014) and Cao et al. (2015). OME requires direct contact between two or more cells, which need to be on a hard surface (Wei et al., 2011). Although OMTs and OMVs could be involved in OME (Remis et al., 2014), it is more likely that these appendages are byproducts of OME or motility (Ducret et al., 2013; Wei et al., 2014). The cell surface-associated proteins TraA and TraB are the two host genetic determinants implicated in OME (Cao et al., 2015). The current model for OME proposed by Cao et al. (2015) is that *M. xanthus* cells physically interact with one another and that TraA–TraA interactions force the opposing membranes into contact, provoking a displacement of water between them which catalyzes outer membrane (OM) fusion. TraB might also interact with TraA to form a functional complex for OM fusion. Fusions are followed by diffusion and exchange of OM contents among neighboring cells. This model of OM fusion is further supported by the finding that TraA/TraB functions as a cell–cell adhesion factor (Vassallo et al., 2015).

*Myxococcus xanthus* lives in a wide range of environments, but it is predominantly found in soils composed of a variety of microbial species and strains (Reichenbach, 1999; Velicer et al., 2014). However, over the years myxobacteriologists have demonstrated that the formation of fruiting bodies is a very selective process and that each fruiting body consists of a single species. This means that myxobacteria are able to discriminate between related and non-related individuals to create social groups. Furthermore, several studies have provided evidence of the presence in natural populations of *M. xanthus* of non-cooperating or exploiting cheaters, which arise from mutations and disrupt or disable multicellular coordination (Velicer et al., 1998; Fiegna and Velicer, 2005; Kraemer and Velicer, 2011). For example, during cooperative predation cheats may consume hydrolyzed products from the prey without the production of hydrolytic enzymes. And there is evidence that the presence of socially defective cheaters during fruiting body formation can reduce group productivity (for instance reducing potential spore production) or can even drive populations to outright extinction (Velicer and Vos, 2009). Several mechanisms may help *M. xanthus* to distinguish self from non-self, thus reducing the risk of exploitation by cheaters and increasing the clonality of fruiting bodies. For instance, the OME process is highly discriminating and it is able to selectively identify kin as exchange partners, which implies the ability of non-identical genotypes to recognize and exclude one another during aggregation and fruiting body formation (Vassallo et al., 2015). Another mechanism that probably contributes to the enrichment of species within fruiting bodies is the specific bacteriocin or antibiotic mediated killing (Smith and Dworkin, 1994). Additionally, it has been suggested that chemotactic responses to some fatty acids, which are enriched during development, play some role in mediating self-recognition during fruiting body formation (Kearns and Shimkets, 2001; Curtis et al., 2006; Lee et al., 2011).

Next, we will review the three best characterized multicellular behaviors of *M. xanthus*: motility, predation, and development.

## Moving Together: Adventurous and Social Motility, Cell Reversals, and Rippling

As mentioned above, *M. xanthus* moves on surfaces by using two complementary flagella-independent motility forms (**Figure 2A**), A-motility and S-motility. Both motility systems, coordinated in space and time, not only facilitate the surface movement of individual cells, but are also essential for the expansion of multicellular swarms, predation and construction of multicellular fruiting bodies (Nan and Zusman, 2011).

A-motility, or gliding motility, drives the movement of single cells at the swarm edges. The A-motile cells glide slowly to explore new environments, change direction through reversal events, and leave behind ECM slime trails that may be followed by other cells (**Figure 2B**). The precise mechanism of A-motility is not completely known. More than 40 genes have been implicated over the years, and for their precise role and regulation readers are referred to reviews by Nan et al. (2014) and Islam and Mignot (2015). There are two main theories proposed for A-motility. The first one, the “helical rotor” model, also called the “crawling snail model,” considers that motors driven by proton motive force (PMF) run along an endless looped helical track driving rotation. Rotation depends not only on PMF but also on an intact MreB cytoskeleton. The gliding complexes are formed by several proteins localized in the cytoplasm, inner membrane, and periplasm (Nan et al., 2010). Some of the gliding motors entering into the ventral region make contact with surfaces, press the gliding surface, deform the cell envelope, and exert force against the polysaccharide slime, propelling the cell forward (Nan et al., 2011, 2013). The second model is the “focal adhesion” mechanism (Balagam et al., 2014; Islam and Mignot, 2015), in which it is proposed that the inner membrane and periplasmic components of the multi-protein cell envelope complexes (Glt complex), attached to a PMF-driven motor (Agl complex), form the Agl-Glt apparatus that moves along the helical track within the cell (Luciano et al., 2011; Agrebi et al., 2015). Once the trafficking complexes engage the outer membrane components, the entire Glt apparatus becomes fixed relative to the substrate via slime, forming a focal adhesion site (Mignot et al., 2007). The continual trafficking of the fixed complex along the helical track propels the cell forward (**Figure 2C**).

Regardless of the model, the role of ECM in facilitating A-motility is unquestionable and it has been demonstrated that it facilitates cell adhesion to the underlying substrate during bacterial surface motility (Ducret et al., 2012). This slime is a self-deposited sugar polymer of unknown composition also containing OM materials from cells that may be deposited in the slime trails during single cell motility (Ducret et al., 2012, 2013). These trails begin at the lagging end of each cell and lengthen as the leading end of the cell advances. Migration of other cells through these trails promotes the formation of dense regions of aligned cells and favors intimate cell–cell contacts (Woglemuth et al., 2002; Yu and Kaiser, 2007), contributing to the occurrence of well-organized pattern networks in these areas (**Figure 2B**). In fact, it has been recently proposed that gliding slime plays an important role in *M. xanthus* social behavior via stigmergic regulation (Ducret et al., 2013). The argument for this proposition is that slime is the physical manifestation of the environment (stimulus) that contributes to the expansion of the community, and continuous traffic increases the amount of slime produced, resulting in additional recruitment of cells migrating along these trails (Gloag et al., 2015). Furthermore, it has been postulated that this trail-following behavior could be similar to the social organization of ants, which is mediated by antennae-borne chemosensory systems (CSS; Kearns and Shimkets, 2001). Coincidentally, one of the foraging pheromones used by ants is 1, 2 diolein (dioleoyl glycerol), a derivative of the lipid phosphatidylethanolamine. It is known that some fatty acids purified from *M. xanthus* cell membranes behaves as a chemoattractants during development (Kearns et al., 2001), so it is likely that lipid chemotaxis is involved in directed movements through the trails. In fact, one abundant ECM-protein (FibA) is important for tactic behaviors toward lipids (Kearns et al., 2002; Bonner et al., 2006). Also, Ducret et al. (2013) have suggested that slime-embedded OM materials or OMVs could contain signals that would promote specific recognition, facilitating trail following and helping colony expansion. Recently, Balagam and Igoshin (2015) have proposed that *M. xanthus* cells use a slime trail-following mechanism to form cell clusters similar to those described for *P. aeruginosa* (Zhao et al., 2013). They suggest that slime trails influence the motility of kin cells that encounter these trails, resulting in them following and further strengthening the trails.

S-motility, or twitching motility, is characterized by the swarming movement of large cell groups and it is stimulated by cell–cell proximity. This motility is crucial for both fruiting body formation (Shimkets, 1986a,b) and cooperative predation (Pérez et al., 2014). Fibrils, which are part of the ECM and form a heterogeneous coat around the cell surface (**Figure 2D**), the lipopolysaccharide (Bowden and Kaplan, 1998), and the retractile type IV pili (T4P; **Figure 2E**) are the extracellular components associated with this type of cooperative motility (Kearns and Shimkets, 2001). Fibrils are thick, flexible structures, mainly composed of a small protein fraction and a particular exopolysaccharide (EPS) that contains glucosamine, galactose, rhamnose, and xylose (Behmlander and Dworkin, 1994a,b; Lu et al., 2005). They make up a malleable meshwork that bundles adjacent cells together and maintains cellular cohesion (**Figure 2F**). They are involved in the activation of the S-motility motor through cell proximity (Yang et al., 2000; Pelling et al., 2005). EPS also exhibits lubricating properties that alleviate the force generation requirements on the lead cell, making coordinated social motility possible (Gibiansky et al., 2013). This EPS also provides chemical signals to guide the two motility systems (Kearns and Shimkets, 2001; Ducret et al., 2013). T4Ps located at the leading cell pole anchor themselves to the carbohydrate portion of the EPS present on neighboring cells (or on slime trails left by A-motile cells) and propel the cell by cycles of extension, attachment and retraction (**Figure 2F**) (Wall and Kaiser, 1999; Skerker and Berg, 2001; Li et al., 2005; Mauriello et al., 2010; Chang et al., 2016). To reverse direction, bacteria disassemble the T4P apparatus on one pole and reassemble it at the other one.

In the swarms, cells are constantly moving, interacting with one another, and reversing their direction in a coordinated manner. In fact, cell reversals and coordination of the two motility systems are needed to achieve the directional movement required for cellular aggregation to form fruiting bodies (Mauriello et al., 2010). These periodical reversal events are timed by a feedback oscillator involving the Frz (frizzy protein) signal transduction cascade, a CSS also called the pacemaker (Mignot et al., 2005; Leonardy et al., 2010; Kaiser and Warrick, 2011; Moine et al., 2014; Guzzo et al., 2015). The mechanism by which the Frz system regulates the timing of gliding reversal and the frequency of switching through the small GTPase MglA, its cognate GTPase-activating protein MglB, and the response regulator RomR has been recently clarified (Bulyha et al., 2011; Kaimer et al., 2012; Keilberg and Søgaard-Andersen, 2014; Islam and Mignot, 2015; Nan et al., 2015). The Frz system has also been proposed as a novel mechanism for coordinating cell movement through cell–cell contact (Mauriello et al., 2009). Moreover, Kaiser and Warrick (2014) reported that the A-motility protein CglB forms protein–protein contacts that may be the signal required to build *M. xanthus* swarms and to synchronize the pacemakers of the connected cells. In addition to the Frz system, motility is also regulated by other CSSs (Kirby, 2009; Moine et al., 2014), which suggests that motility can be chemotactic (Zhang et al., 2012b). Other evidence, such as treatment with attractant lipids or toxic compounds that leads to changes in the reversal periods, supports that hypothesis (Kearns and Shimkets, 1998).

**FIGURE 3:**
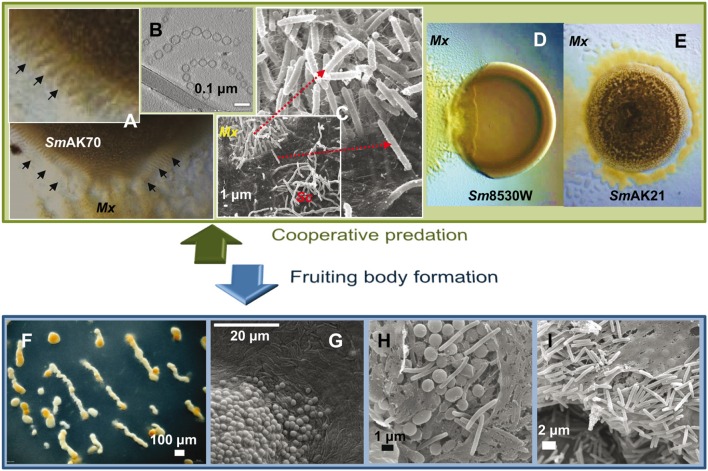
***Myxococcus xanthus* multicellular behaviors.** **(A)** *M. xanthus* DK1622 coordinated movements (rippling) induced by *Sinorhizobium meliloti* AK70 (*Sm*AK70) during predation. **(B)** OMV chains cryofixed and visualized by transmission electronic microscopy. OMVs contain multiple hydrolytic enzymes and secondary metabolites indicating an important role in killing and lysis of prey. **(C)** Scanning electron micrographs of the interface DK1622 (*Mx*) versus *Streptomyces coelicolor* M45 (*Sc*) cells after 96 h of incubation. The magnified picture shows the intense ECM connection between the attacking cells. **(D)** Frontal attack strategy: *M. xanthus* DK1622 (*Mx*) versus a non-mucoid colony of *S. meliloti* 8530W (*Sm*8530W). **(E)** Wolf pack attack strategy: *M. xanthus* DZ2 *(Mx)* versus a mucoid colony of *S. meliloti* AK21 (*Sm*AK21). **(F)** *M. xanthus* DZF1 fruiting body formation after 72 h on starvation medium. **(G–I)** Scanning microscopy of *M. xanthus* DZF1 fruiting body showing the round spores and the surrounding peripheral rods. **(H,I)** Within the fruiting bodies, the myxospores are firmly bound together by a cohesive ECM. Picture **(B)** has been adapted from Remis et al. (2014) with permission, copyright 2013 Society for Applied Microbiology and John Wiley & Sons Ltd.

In the presence of cell debris, peptidoglycan, or many other macromolecules (Shimkets and Kaiser, 1982; Sager and Kaiser, 1994; Welch and Kaiser, 2001; Berleman et al., 2006; Pérez et al., 2014), *M. xanthus* cells organize their movement into accordion-like waves (**Figure 3A**), which look similar to ripples in water (Stevens and Søgaard-Andersen, 2005; Sliusarenko et al., 2006). During rippling, each wave crest oscillates back and forth with no net displacement, although individual cells change position. When two waves collide, cells in one wave penetrate the opposing wave by one cell length, followed by cell reversals. Experimental and theoretical studies of rippling behavior indicate that these moving patterns can be produced by a side-to-side signaling between two cells that may cause one of the cells to reverse, by physical interactions that cause the cell to locally align, and by an internal biochemical oscillation system (Igoshin et al., 2001, 2004; Sliusarenko et al., 2006; Zhang et al., 2012a).

Finally, motility is also critical for the aforementioned OME. In fact, the first proteins known to be substrates for transfer by OME are the A-motility protein CglB and the S-motility protein Tgl (Hodgkin and Kaiser, 1979; Nudleman et al., 2005). Later, several investigations have concluded that although OM protein exchange is not restricted to motility proteins (Wei et al., 2011; Pathak et al., 2012; Vassallo et al., 2015), at least one partner (either donor or recipient) needs to be motile (Dey and Wall, 2014; Cao et al., 2015). The role that motility plays in OME has not been clarified, although it most likely facilitates the proper cell–cell alignments and contacts that lead to exchange, or it may participate in incorporating OM materials into the slime polymer when cells follow the slime trails (Ducret et al., 2013; Wall, 2014).

## Killing and Feeding Together: Cooperative Lysis and Group Predation Strategies

During vegetative growth, *M. xanthus* can grow as a saprophyte on dead organic matter by decomposing degradable polymers or prey upon a variety of Gram-negative and Gram-positive bacteria, as well as fungi (Morgan et al., 2010; Müller et al., 2014; Pérez et al., 2016). This activity is also a multicellular process and the efficiency of its feeding style seems to be density-dependent (Rosenberg et al., 1977). Although individual *M. xanthus* cells are able under certain circumstances to catch prey following a cell-to-cell attack (McBride and Zusman, 1996), the usual feeding strategy is cooperative predation (Pérez et al., 2016). However, it is unknown whether *M. xanthus* shows a cell density-dependent genetic expression of predatory enzymes or if, conversely, these enzymes are expressed constitutively. In the latter case, high cell density would simply facilitate predation by increasing the concentration of extracellular lytic factors (Berleman and Kirby, 2009).

The first step in predation involves *M. xanthus* motility because predator cells require close proximity to the prey (McBride and Zusman, 1996). Predatory groups (**Figure 3C**) actively swarm toward the prey using the two motility systems described previously. The roles in predation of the two motility systems are imprecise, but it seems that both A- and S-motility engines are required for efficient predation (Pham et al., 2005; Berleman et al., 2006; Berleman and Kirby, 2009; Pérez et al., 2014). Contact with prey cells stimulates reversals that are responsible for individual *M. xanthus* cells becoming trapped in prey micro-colonies until prey-cell lysis is complete (Keane and Berleman, 2016). Additionally, at higher cell densities, contact with the prey also yields rippling behavior. However, the role of rippling in predation remains to be clarified. Until recently, rippling was accepted as a critical predatory behavior that serves to maximize predation efficiency and nutrient scavenging (Berleman et al., 2006, 2008). Nevertheless, rippling is not always necessary for predation because it neither helps to overcome the physical and chemical barrier conferred by the prey nor improves prey lysis (Pérez et al., 2014).

The attack strategy of *M. xanthus* seems to depend on the nature of the prey. By studying predation on different *Sinorhizobium meliloti* strains, it has been shown that this myxobacterium can follow two predatory tactics that depend on the presence of the rhizobial EPS galactoglucan. The strategy used by *M. xanthus* against laboratory strains of *S. meliloti* that lack galactoglucan is identical to that previously described for other prey (Berleman et al., 2008). This strategy resembles a frontal attack (**Figure 3D**), where groups of predators progressively penetrate the prey colony and lyse the cells (Berleman et al., 2008; Pérez et al., 2011, 2014). The other strategy fits well with the traditional definition of *M. xanthus* predation strategy, referred to as wolf-pack attack (**Figure 3E**). In this case, *M. xanthus* cells surround the prey colony and ripple before killing and lysing the field-isolated galactoglucan-holding *S meliloti* strains (Pérez et al., 2014).

During predation, *M. xanthus* swarms secrete a plethora of hydrolytic enzymes, antibiotics, and other secondary metabolites that lyse the prey cells, releasing a pool of hydrolyzed products into the extracellular milieu, which are consumed by the myxobacteria (Sudo and Dworkin, 1972; Wenzel and Müller, 2009; Xiao et al., 2011; Evans et al., 2012). How *M. xanthus* protects itself from lysis by its own extracellular digestive enzymes is unknown, but it has been suggested that the ECM could play a protective role, because no mutants failing to produce any propulsive slime have been isolated despite the efforts of different laboratories (Kaiser, 2015). Molecules associated with predation and prey nutrients will both tend to travel away from the generating cell, providing a chance for exploitation by others and increasing the dilution by diffusion (Whitworth, 2011; Mendes-Soares and Velicer, 2013). In this predation strategy, cheating is a potential problem because the predator strains generate a publicly accessible resource, which cheats might consume at the expense of cooperative strains (Evans et al., 2012). As mentioned above, *M. xanthus* produces OMVs in large quantities (**Figure 3B**) and their role in multicellular behavior is still under research (Whitworth, 2011; Remis et al., 2014; Keane and Berleman, 2016). However, proteomics studies of OMVs have demonstrated that they contain multiple hydrolytic enzymes and secondary metabolites associated with antibiotic activities (Kahnt et al., 2010; Berleman et al., 2014; Remis et al., 2014). Moreover, functional studies also indicate that OMVs play an important role in predation (Remis et al., 2014). The packaging of multiple predatory molecules within the OMVs, which deliver the lethal cocktail to the prey cells, slows the transport rate of lytic factors away from the immediate vicinity of the producing organism and reduces the risk of potential competition or exploitation by cheats, increasing the predation efficiency (Whitworth, 2011; Evans et al., 2012; Berleman et al., 2014).

The multiplicity of activities and functions associated with group predation implies a considerable number of signal-transduction processes for detecting and tracking prey, and for coordinating all the metabolic pathways involved in neutralizing and lysing the prey, to which the uptake and incorporation into the myxobacterial metabolism of the released nutrients must be added. Although this social behavior was first described 75 years ago, most of these systems remain to be elucidated. This is mainly due to the fact that most researchers have concentrated their interest on the developmental cycle. Moreover, *M. xanthus* is not an obligate predator since it grows well in a rich medium. Consequently, studies on predation are lagging far behind those of other predators such as *Bdellovibrio* (Pérez et al., 2016). It is known that this interaction has some consequences for the prey. For instance, *S. meliloti* AK21 cells respond to the approaching myxobacteria by producing EPS and retreating (Pérez et al., 2014). It is also known that *M. xanthus* induces the formation of spore-filled megastructures in *B. subtilis* (Müller et al., 2015), and production of antibiotics and differentiation in *Streptomyces coelicolor* (Pérez et al., 2011). These results support the idea that predator–prey interaction can increase the production of secondary metabolites and, consequently, co-culture with the prey may be a strategy worth considering in biotechnology research on the natural products of myxobacteria. Many questions need to be answered in the near future, such as what the real ecological consequences of *M. xanthus* predation are on natural environments, how the prey induces the predatory enzymes or secondary metabolites in *M. xanthus*, which other natural barriers besides the EPS galactoglucan determine the predation strategy of the myxobacteria, how predators detect the prey, why contact with the prey is necessary for predation, and how predators maximize nutrient uptake.

## Surviving Together: Division of Labor, Fruiting Body Formation, and Intra-/Inter-cellular Signaling

When nutrients become limited, the vegetative spread of the myxobacteria is constrained and the population initiates a developmental program that culminates with the formation of multicellular, spore-filled fruiting bodies (**Figures 3F,G**). Fruiting body formation requires a solid surface to allow motility, a threshold population density, recognition at the cellular level of the nutrient downshift, and a complex series of inter- and intracellular signaling that proceeds in distinct morphological stages separated in time and space (Diodati et al., 2008; Higgs et al., 2014; Rajagopalan et al., 2014).

The first signs of development are detectable after 4–6 h of starvation, when cells aggregate to form small aggregation centers. Over the course of the next several hours, these initial foci may disintegrate, merge or increase in size to become larger aggregates. Shortly after reaching the aggregation center (6–12 h), these cells show a coordinated sequence of three highly characteristic morphological changes, including formation of large intracellular lipid bodies, cellular morphogenesis into spherical prespore cells, and formation of a thick, multilayered spore envelope. As the aggregation centers accumulate more cells, they eventually become mound-shaped. By 24 h the aggregation process is complete and each nascent fruiting body contains approximately 10^5^–10^6^ densely packed cells. Inside the fruiting bodies, the vegetative rod-shaped cells undergo morphological and physiological differentiation into spherical myxospores (**Figures 3G,H**). Spore maturation is finished approximately 72 h after the onset of starvation. Within the fruiting bodies the myxospores are firmly bound together by the cohesive ECM (**Figures 3H,I**), hence, upon germination, the whole population creates a new swarm (Diodati et al., 2008).

The number of different cell types that occur in a group can be compared to the number of castes in eusocial insect colonies, in which the different group members specialize at different roles. The total number of cells in those groups is perceived as one of the factors that contributes to and correlates with group complexity (Strassmann and Queller, 2010; Fisher et al., 2013). As mentioned above, the *M. xanthus* developmental monoclonal population segregates into one of three subpopulations that show division of labor (**Figure 1**): 10% of cells differentiate into spores that are produced within multicellular fruiting bodies and are resistant to heat, desiccation, and nutrient deprivation (O’Connor and Zusman, 1991a); 30% of cells differentiate into peripheral rods (a persister-like state) that remain on the exterior of the fruiting body (O’Connor and Zusman, 1991a,b); and the remaining cells undergo lysis by PCD (Wireman and Dworkin, 1977; Nariya and Inouye, 2008).

The complex cellular differentiation from rod-shaped vegetative cells into round spores involves remodeling of the cell envelope, the synthesis of the rigid spore coat (Müller et al., 2012), the formation of a two-chromosome complement (Tzeng and Singer, 2005), and the synthesis of spore-specific lipid components (Ring et al., 2006). During sporulation, *M. xanthus* cells undergo massive reprogramming of their gene expression patterns (Müller et al., 2010; Higgs et al., 2014) and extensive metabolic rearrangements. Intracellular lipid bodies are probably used to fulfill these cellular metabolic requirements. In fact, lipid bodies gradually disappear during spore maturation until they are entirely used up when the cells have completed the differentiation process (Ring et al., 2006).

Peripheral rods are a discrete subpopulation of cells that move around and between fruiting bodies scouting for food. It has been proposed that they have evolved to take advantage of low levels of nutrients which are insufficient to either promote growth or to induce the germination of the spores inside the fruiting bodies (O’Connor and Zusman, 1991a). For this reason, this subpopulation is considered to function as persistent cells which do not undergo cell division but are likely ready to respond to any sudden increase in nutrients (O’Connor and Zusman, 1991a; Higgs et al., 2014). Returning to the example of insect colonies, in the case of *M. xanthus*, these sterile cells could represent a case of extreme altruism, as happens with sterile workers in eusocial insects, sacrificing any opportunity of reproduction in order to help others. However, and although first described as altruistic (Zahavi and Ralt, 1984), many myxobacteriologists consider that both sporulation and differentiation into peripheral rods are in fact two different survival strategies which, combined, confer *M. xanthus* with a more complete resistance to adverse conditions (Shimkets, 1999; Kaiser et al., 2010; Mauriello et al., 2010; Zhang et al., 2012b). Even though peripheral rods superficially resemble vegetative cells (**Figures 3C,I**), different analyses have shown that these cells appear to become hyperpiliated, do not significantly accumulate extracellular polymeric substances, express markers that clearly distinguish them from vegetative cells, and express different genes from those of sporulating cells (O’Connor and Zusman, 1991a; Higgs et al., 2014). Peripheral rods, while experiencing the same starvation process, do not form lipid bodies, are unable to form fruiting bodies, and do not differentiate into spores (Hoiczyk et al., 2009). One model proposed to explain the differentiation into peripheral rods suggests that they may be produced by cells that do not make sufficient end-to-end contacts to efficiently exchange the C signal (Julien et al., 2000). A different model proposes that peripheral rods may arise from cells that fail to accumulate sufficient levels of the developmental transcriptional regulators MrpC and its target FruA (Lee et al., 2012).

The third type of cell fate during development is PCD. The onset of cell lysis immediately precedes, or is concomitant with, the onset of aggregation, but it likely continues during maturation of the fruiting bodies, to provide nutrients that allow spore differentiation. Some authors consider that cell lysis could also play a role in aggregation (Lee et al., 2012). It is still unknown whether cells undergo an altruistic PCD process or if it is a product of intra-swarm competition. Two different mechanisms of PCD have been proposed. One of them involves the production of autocides, characterized as a mixture of fatty acids and phosphatidyl ethanolamine that permeabilizes the cells and ultimately leads to lysis (Varon et al., 1984, 1986; Gelvan et al., 1987). The other hypothesis is based on the toxin-antitoxin system, consisting of MazF (toxin) and MrpC (antitoxin; Nariya and Inouye, 2008). It should be noted that some experiments have demonstrated that this latter mechanism does not function with all *M. xanthus* strains (Lee et al., 2012; Boynton et al., 2013). The evolution and maintenance of cell lysis as an altruistic and cooperative process is possible only in groups of closely related individuals. Although the production of public goods by the lysis of the majority of cells benefits the community, some individuals can exploit this situation by using nutrients without contributing toward their production. Such individuals have a fitness advantage, as they do not utilize their own resources, yet they enjoy the benefits. This leads to an increase in the number of these social cheaters in the population. Exploiter populations compete with cells that cooperate and, eventually, the entire social structure will collapse as nutrients will not be available at sufficient levels (Cao et al., 2015; van Gestel et al., 2015). Consequently, this process requires a mechanism to discriminate kin from non-kin that could be mediated by OME (Cao et al., 2015). By this system, cells that belong to the same *traA* recognition group are mutually immune to bacteriocin-mediated killing, whereas cells from different recognition groups are able to kill each other (Pathak et al., 2013).

In addition to the different cell fates described above, phase-variation and cell clustering also play different roles during development. *M. xanthus* undergoes phase variation to produce non-pure yellow or tan colonies, where both variants can switch from one to the other (Laue and Gill, 1994). Phase variation in *M. xanthus* affects swarming, texture of the colony, pigmentation, fruiting body formation, and sporulation (Meiser et al., 2006). During growth, wild-type yellow variants (WTY) accumulate at the colony edge and surround the slower swarming wild-type tan variants (WTT; Dziewanowska et al., 2014). WTY cells accumulate DKxanthene, a secondary metabolite that confers the characteristic yellow color (Meiser et al., 2006), and the antibiotic myxovirescin, which are needed for sporulation and predation (Xiao et al., 2011). WTT cells, which produce elevated levels of iron acquisition systems, fail to form mature fruiting bodies and also produce fewer spores (Furusawa et al., 2011). Nevertheless, WTT cells contribute disproportionately to the population of spores, although the presence of yellow cells is necessary since they provide some factor (maybe DKxanthene or myxovirescin) that the tan cells need in order to produce viable spores (Meiser et al., 2006). This mutual dependence might guarantee their survival. Transcriptomic analyses of WTY and WTT cells have initially revealed that less than 1% of the *M. xanthus* genome is devoted to this process, with only 41 genes differentially regulated during phase variation (Furusawa et al., 2011). Further global studies with WTY, WTT and three tan mutants have raised this number to more than 200 genes (∼2.8%; Dziewanowska et al., 2014).

Cell cluster subpopulations represent an additional source of heterogeneity in *M. xanthus*, consisting of cells that are found in tight aggregates within the swarm (Lee et al., 2011, 2012). During growth, cell clusters may simply better weather changes in the environment, while during development they could serve as a platform upon which aggregating cells build fruiting bodies (Curtis et al., 2007), or as a determinant to control appropriate spacing between fruiting bodies (Xie et al., 2011).

Several hypotheses regarding the benefits of fruiting body formation have been proposed. First, it may represent a budding strategy that reduces local competition among kin ensuring the prevalence over other species in their new habitat, allowing small groups of kin to disperse together and thereby retain the benefits of kin association during cooperation (Gardner and West, 2006). Second, sporulation within fruiting bodies might also produce higher quality spores than individualistic sporulation would (Wireman and Dworkin, 1977). Third, the ECM in which spores are embedded (**Figures 3H,I**) may protect them against various external threats (e.g., predation or anti-competitor compounds) or abiotic stresses (Ward and Zusman, 2000), and may improve germination efficiency maintaining a high density of packed spores (Shimkets et al., 2005). Finally, it has been suggested that the complexity of all these multicellular behaviors during myxobacterial development ultimately helps to maintain their special predatory lifestyle and to support the growth on large and insoluble molecules (Keane and Berleman, 2016).

In *M. xanthus*, starvation triggers the stringent response, which involves production of the second messenger (p)ppGpp (Singer and Kaiser, 1995; Boutte and Crosson, 2013). The two main intercellular signaling programs (A and C) depend on (p)ppGpp (Manoil and Kaiser, 1980a,b; Harris et al., 1998; Crawford and Shimkets, 2000a,b), and accumulation of (p)ppGpp is both necessary and sufficient for *M. xanthus* to enter into the developmental cycle (Shimkets, 1999). From that point onward, protein synthesis aimed at vegetative growth switches to controlled proteolysis for self-supply and specific protein synthesis for fruiting body and spore formation (Diodati et al., 2008). More than 2000 genes (∼30% of the genome) are differentially expressed in developing cells (Giglio et al., 2011), which are sequentially activated, and some of the gene products are even spatially localized (Rajagopalan et al., 2014). Moreover, 1486 genes are significantly upregulated during an artificial chemically induced sporulation process (Müller et al., 2010). The complex gene regulatory network controlling this process comprises a large number of genes belonging to the four main signal-transduction systems described in bacteria: one-component systems, two-component systems (TCS), extracytoplasmic function sigma factors, and serine/threonine protein kinases (STPK)/phosphatases (Muñoz-Dorado et al., 2012). According to Rajagopalan et al. (2014), this gene regulatory network includes three modules, with starvation, intracellular (p)ppGpp and extracellular A and C signals providing inputs to these regulatory modules. The first module consists of a cascade of enhancer binding proteins (EBPs) that functions by activating early developmental genes (Caberoy et al., 2003). The second module, known as the Mrp module, depends on EBPs and the A signal, and includes the *mrpAB* operon and the *mrpC* gene. This module governs the accumulation of MrpC and its truncated form MrpC2. The third module, the FruA module, depends on MrpC and the C signal. During this module, MrpC induces the expression of *fruA* (Ueki and Inouye, 2003) and acts as an antitoxin to control PCD in some strains (Nariya and Inouye, 2008). It seems that FruA and MrpC2, along with the C signal, regulate the expression of the *dev* operon, which is involved in spore formation (Rajagopalan et al., 2014).

The intercellular signaling involves, along with the A and C signals, three other signals called B, D, and E signals (Kaiser, 2004). All of them are essential to successfully completing the developmental cycle. Out of the five signals, A, C, and E are the ones that have been chemically characterized.

The quorum-sensing A signal consists of a set of peptides and amino acids that are released into the extracellular milieu by proteases (Kuspa et al., 1986, 1992; Plamann et al., 1992). Interestingly, the amino acid composition of the A signal is different from that of the average composition of the cell and spore proteomes. Although it seems counterproductive to use nutrients as a signal of nutrient scarcity, the evolutionary advantage may rely on the ability of the cells to distinguish between real nutrient-poor conditions and dishonest signals coming from cheating *M. xanthus* cells, thus avoiding the population entering into development when food is available (Whitworth et al., 2015). (p)ppGpp leads to the activation of early developmental genes and the secretion of protease activity that generates the A signal. This extracellular signal appears to be unique among bacteria, but plays a role analogous to that of quorum sensing in other bacteria, acting as an indicator of population density. The A signal controls the entry into development if the population density and nutrients are both sufficient. This ensures that the swarm initiates development before any nutrient becomes too scarce to synthesize the new proteins necessary for fruiting body and spore formation (Konovalova et al., 2012b). The genetic operon involved in the production of the A signal consists of five genes called *asgA*, *B, C, D*, and *E*. Nutrient scarcity is detected by the histidine kinase AsgA, which promotes the phosphorylation of AsgB, leading to the expression of genes responsible for the production of the A signal (Konovalova et al., 2012b). Although the triggering genes for A-signal production have been characterized, so far neither the proteases nor the receptor proteins have been identified. The A signal is perceived by a two-component system named SasS/SasR. The histidine kinase SasS detects the amino acids and peptides that are released into the extracellular milieu and the response regulator SasR activates the expression of A-signal dependent genes (Kaiser, 2004).

On the other hand, the C signal, which coordinates aggregation later in development, depends on the gene *csgA* (Kim and Kaiser, 1990a,b; Konovalova et al., 2010). A-motility is necessary for its transmission, so that aligned cells can move to contact one another through their poles, where the C signal is located. The gene *csgA* encodes a 25-kDa protein (Hagen et al., 1978; Shimkets et al., 1983). According to the current model, the 25-kDa CsgA protein is secreted and remains anchored to the OM. Once there, the protein is processed by the protease PopC, resulting in a smaller protein (17 kDa), which functions as the C signal (Konovalova et al., 2012a). However, to date no molecular receptor for the processed C signal has been found. Moreover, several studies have demonstrated that CsgA is an inner membrane protein, not an outer membrane protein, with certain homology to the short-chain alcohol dehydrogenase family of proteins (Simunovic et al., 2003; Kahnt et al., 2010; Bhat et al., 2011). To explain these inconsistencies, a novel hypothesis about the C-signaling mechanism has been proposed by Boynton and Shimkets (2015). They have demonstrated that the 25-kDa protein has phospholipase C-like activity and proposed that *M. xanthus* cells use this activity to convert inner membrane phospholipids into triacylglycerol, which eventually provokes cells to shorten and become round for spore formation, creating lipid bodies during the process. In spite of the efforts that have been made to explain the mechanism of C-signal transmission in *M. xanthus*, further research and new data are required to establish a conclusive model.

The transmission of the C-signal triggers three decisive developmental processes and ensures that they occur at the appropriate time and location. The C signal first induces the process of rippling, then aggregation, and finally sporulation (Kaiser, 2003). Each of the three processes is initiated after surpassing a different threshold of the C signal. The C signal is synthesized during growth, but at a very low concentration. The number of C signal molecules per cell increases as development progresses, stimulated by a positive feedback mechanism that increases expression of *cgsA* (Gronewold and Kaiser, 2001; Kaiser, 2015). At low levels of C signal (10–20% of maximum developmental levels), 3 h after nutrient depletion, rippling is induced (Kim and Kaiser, 1991; Li et al., 1992; Boynton and Shimkets, 2015). After 8–18 h, at medium levels of signal, each cell harbors several hundred C signal molecules, and aggregates begin to form. Sporulation is triggered only after C signal production in the cells is high and it has been transmitted long enough. Then, C signal concentration peaks and induces sporulation. High levels of C signal are only reached inside the fruiting bodies, where cell density is high enough. As mentioned above, peripheral rods harbor lower levels of C signal and for this reason these cells neither aggregate nor sporulate (Kaiser, 2003).

The expression of genes depending on C signal starts 6 h after starvation (Konovalova et al., 2010, 2012b). When the C-signal is transmitted, a phosphorylation cascade is activated inside the cell, which results in phosphorylation of the response regulator FruA by an unidentified histidine kinase. Phosphorylated FruA becomes active and initiates two signal-transduction pathways (Kaiser, 2004). One of them, activated through low levels of C signal, triggers aggregation by modifying gene expression of the *frz* genes, which regulates the reversal frequency of the swarm. This modification causes cells to travel in circles and end up forming aggregates. The second signal-transduction pathway is activated when the C signal reaches high levels and has been transmitted long enough. Activation of this pathway triggers the transcription of the *dev* operon, which initiates and controls the process of sporulation (Rajagopalan et al., 2014).

Finally, the E-signal is a combination of the branched chain fatty acid iso-15:0 and the diacylmonoalkyl ether lipid TG1. Several studies indicate that these lipids are involved in signaling during fruiting body formation (Bhat et al., 2014; Ahrendt et al., 2016).

Since the adaptive capacity of *M. xanthus* is the result of its collective adjustment to the two social stages of its life cycle (**Figure 1**), an extensive co-evolution is expected (Velicer et al., 2014). In fact, mutations affecting development often have an impact on predation. The predatory ability of *M. xanthus* is adversely affected by mutations in genes implicated in the early stages of development (*asgA*, *asgC*, *asgE*, *sdeK*, and *csgA*), as well as in genes of the chemotactic *frz* system, which modulates motility and development (Pham et al., 2005; Berleman et al., 2006, 2008). Conversely, genes required at late stages of development (*dev*RS, *MXAN4406*, and *phoP4*) do not seem to alter predation (Pham et al., 2005). The demonstration that two EBPs (MXAN_4899 and HsfA), which are involved in motility and fruiting body formation, also regulate the production of secondary metabolites implicated in predation is a clear example of different multicellular processes being linked to transcriptional levels and of these bacteria using exceptional mechanisms for the integration of development, predation, and motility (Volz et al., 2012). Additionally, the two chaperones GroEL1 and GroEL2, which are localized in OMVs, may be involved in the proper folding of proteins responsible for the coordination of both multicellular processes (Keane and Berleman, 2016). In fact, GroEL1 is involved in development (Li et al., 2010), and GroEL2 is needed for the synthesis of some secondary metabolites and efficient predation (Wang et al., 2014).

## Genomes and Proteomes of Myxobacteria and Multicellularity

In the last 10 years, 25 myxobacterial genomes have become available (NCBI database, January 2016). Except for the family *Anaeromyxobacteraceae*, whose members do not form fruiting bodies and have genomes of around 5 Mb, the rest of the species form fruiting bodies and have the largest genomes described among prokaryotes, ranging from 9 to 14.8 Mb (**Supplementary Table S1**). Since myxobacteria show multicellular behaviors, it is tempting to speculate that their large genomes and large proteomes are necessary for their particular way of life. Indeed, it has been noted that bacteria with multicellular forms typically have large proteomes (Aravind et al., 2009), in contrast to eukaryotes, in which larger proteome size does not necessarily imply a multicellular morphology. In fact, two of the largest eukaryotic proteomes are those of unicellular organisms such as ciliates (Eisen et al., 2006) and *Trichomonas* (Carlton et al., 2007). Comparative transcriptomic analyses performed at different pHs with the myxobacterium *Sorangium cellulosum* So0157-2 (isolated from alkaline soil), which holds the largest bacterial genome reported to date, demonstrate complex expression patterns under fluctuating environmental conditions (Han et al., 2013). Their results indicate that this bacterium has undergone an extraordinary genome expansion via horizontal gene transfer and gene duplication to enable it to survive in difficult environmental conditions. They suggest that bacteria living in complex and changing environments have more internal and external opportunities to expand their genomes. All these data suggest that a large genome is the result of developmental complexity and adaptation to variable environments (Han et al., 2013).

Because of the multiple origins of multicellular behaviors among bacteria, it is plausible to think that they have little in common. However, comparative genomics has revealed certain shared features at the molecular level (Aravind et al., 2009). Several studies have examined the independent transitions to multicellularity in social bacterial lineages. For example, a comparison of differentiated multicellular cyanobacteria with their undifferentiated multicellular and unicellular relatives and of the multicellular actinobacterium *S. coelicolor* with its unicellular relatives both revealed large increases in genes involved in signal transduction and transcriptional regulation. A large fraction of these additional genes have appeared as a result of gene duplication events (Rokas, 2008). This is consistent with the expansion of myxobacterial genomes having arisen largely through gene duplications of specific gene families, particularly those involved in cell signaling and signal transduction, which are likely to function in cell–cell interactions to maintain multicellularity and in response to changing environmental conditions (Goldman et al., 2006; Schneiker et al., 2007; Pérez et al., 2008; Huntley et al., 2011). In fact, Goldman et al. (2006) found more than 1500 duplications that probably occurred during the transition to multicellularity and they suggested that cell–cell signaling and regulatory genes underwent 3–4 times as many duplications as would be expected by probability. It has been stated that *M. xanthus* metabolic genes have been acquired via horizontal gene transfer, whereas signaling genes arise mainly by duplication and divergence (Goldman et al., 2006).

A thoughtful comparative genomic study by Huntley et al. (2011) of four developing (*M. xanthus*, *Stigmatella aurantiaca*, *Haliangium ochraceum*, and *S. cellulosum*) and one non-developing (*Anaeromyxobacter dehalogenans*) myxobacterial species revealed that 1052 genes were conserved in all five species, corresponding to the *Myxococcales* core genome. As many as 85% of the core genes have functions inferred, which are distributed in different clusters of orthologous groups. Interestingly, 5% of these genes correspond to the category of signal transduction. About 425 genes were conserved between the developing myxobacteria, suggesting that this set represents the signature genes that encode the functions needed for fruiting body formation. Most of those signature genes have orthologs outside the *Myxococcales*, but, interestingly, the *Myxococcales* specific genes are mainly involved in signal transduction. One-component systems are underrepresented in the myxobacterial genomes, following the deltaproteobacteria trends (Goldman et al., 2006; Huntley et al., 2014). However, TCSs increase linearly with genome size, in contrast to the overrepresentation of this type of signal-transduction mechanism in all the non-myxobacterial deltaproteobacteria genomes (Huntley et al., 2014). The preponderance of orphan TCSs, the presence of many complex clusters, and the atypical architecture domains of many myxobacterial TCSs indicate that these social bacteria have exploited the simple signal transmission of the bacterial TCSs to produce intricate multi-component systems that approach the complexity of eukaryotes (Muñoz-Dorado et al., 2014). Special mention should be given to the abovementioned CSSs, which can be considered as specialized TCSs. Genomic analyses indicate that *M. xanthus* produces an unusual number of chemosensory proteins. Phylogenetic, distribution, genomic organization, and subcellular localization studies have shown that complex behaviors such as social motility, reversal frequency, development and biofilm formation require regulatory apparatus composed of multiple interconnected CSS systems (Moine et al., 2014). In fact, many *M. xanthus* TCSs and CSSs have functions in motility or development (Moine et al., 2014; Muñoz-Dorado et al., 2014). A recent genome-wide study of myxobacterial TCSs suggests that there is a core set of TCS genes involved in the regulation of important functions and another accessory set that varies between genomes. This study also concludes that the individuality of myxobacterial TCS gene sets seems to be primarily due to lineage-specific gene loss, although there is also evidence of extensive acquisition of genes by horizontal gene transfer and gene duplication (Whitworth, 2015). It is also notable that the *M. xanthus* genome holds a subgroup of 52 homologs of EBPs, 18 of which have been demonstrated to be involved in development (Giglio et al., 2011). Myxobacterial genomes are also enriched in extracytoplasmic function sigma factors, some of which are implicated in carotenoid biosynthesis and metal homeostasis, while others participate in social behaviors such as motility and development (Gómez-Santos et al., 2011; Han et al., 2013; Abellón-Ruiz et al., 2014; Huntley et al., 2014; Marcos-Torres et al., 2016). However, the most intriguing feature regarding signal-transduction gene expansion is the abundance in fruiting-body forming myxobacteria of STPKs, which represent the main signal-transduction systems in eukaryotes. Pérez et al. (2008) found that other bacterial genomes with social activities also hold many STPKs, suggesting multicellular behavior as the main evolutionary driver for an extensive kinome. Moreover, proteomic analyses carried out in the myxobacterium *S. cellulosum* So ce56, which contains a large kinome, have shown that post-translational phosphorylation plays a particularly important role in the regulation of its complex social lifestyle (Schneiker et al., 2007). The same trend can be seen in *S. cellulosum* So0157-2, where the largest gene family of paralogous genes also corresponds to STPKs, with 508 members (Han et al., 2013). In *M. xanthus*, 13 STPKs are involved in regulating development and motility (Muñoz-Dorado et al., 1991, 2014; Inouye et al., 2008) and another 12 STPKs seem to be implicated in phase variation (Dziewanowska et al., 2014). Although bacterial multicellularity clearly relies on other factors in addition to STPKs, all the data indicate that large kinomes have evolved preferentially to support this lifestyle and that complex signaling and intricate regulatory networks are important for multicellularity.

Huntley et al. (2014) analyzed 95 known *M. xanthus* development-specific genes to study the conservation of the *M. xanthus* genetic program for development in other myxobacteria. Their results indicate that those developmental genes could be part of an ancestral response to starvation rather than being involved in development. The fact that one of the conserved proteins in all myxobacteria is RelA, which is involved in the stringent response, reinforces this hypothesis. The authors also observed that the 95 developmental genes are not highly conserved in the non-developing species *A. dehalogenans*, that they are overrepresented in the myxobacteria species belonging to the suborder *Cystobacterineae*, and that they are not conserved in the suborders *Nannocystineae* and *Sorangineae*. For instance, the developmental MrpC/FruA/C signal transduction pathway is lacking in *A. dehalogenans*, *S. cellulosum*, and *H. ochraceum*. These observations suggest that the myxobacterial genomes display a high degree of plasticity in the genetic programs resulting in fruiting body formation and sporulation that differ significantly in each suborder.

Reflecting its predatory strategy, as much as 8.6% of the *M. xanthus* genome is devoted to encoding enzymes for secondary metabolism (including polyketide synthases and non-ribosomal polypeptide synthetases) and degradative enzymes such as those involved in hydrolysis of peptidoglycan, proteases, and metalloproteases, implicated in depolymerization of the prey macromolecules. An extensive arsenal of natural products with cytotoxic, antibiotic, bacteriocin-like, antiviral, antifungal, insecticidal, antiparasitic, and anti-tumor properties has been isolated and identified in the families *Myxococacceae*, *Polyangiaceae*, *Cystobacteraceae, and Haliangiaceae* which are under research for potential applications in the pharmaceutical and biotechnology industries (Garcia and Müller, 2014a,b,c; Knupp dos Santos et al., 2014). The physiological functions of these bioactive molecules are still unclear, although it has been shown that antibiotics play a crucial role in myxobacterial predation (Xiao et al., 2011; Pérez et al., 2016). Therefore, the idea that these bioactive compounds are used by myxobacteria to prey on other organisms and defend their ecological niches needs to be deeply explored.

Finally, a comparative genomics study of predators and non-predators, in which *M. xanthus*, *S. aurantiaca*, and *S. cellulosum* were included, revealed that the genomes of predators exhibit deficiencies in riboflavin and amino acid biosynthesis whereas they are highly enriched in genes for adhesins, proteases, and other proteins probably used for binding to, processing, and consuming their prey (Pasternak et al., 2013).

## Concluding Remarks and Future Perspectives

Myxobacteria, and in particular *M. xanthus*, are capable of multifaceted social behaviors that maximize the use of resources and their survival by adopting a multicellular lifestyle. They choreograph the behavior of the population to allow cooperative predation of other bacteria and the formation of complex fruiting bodies. Motility (either A- or S-motility) is itself a cooperative behavior that contributes to the social lifestyle of *M. xanthus* and is essential for both predation and fruiting body formation. Many elements and contexts contribute to myxobacterial multicellularity. The ECM is of central importance to the correct assembly and maintenance of the biofilm, and to helping cells to stay in contact with the substrate on which they move. The existence of a network of OMVs and OMTs also helps to physically interconnect and bind cells during cooperative behaviors within these biofilms. The proximity of the cells not only facilitates cell–cell signaling, but it is also essential for OME, a mechanism that enables transfer between two or more cells of OM lipoproteins, proteins, lipids, or lipopolysaccharide. OME between healthy cells and damaged cells enables motility, development, antibiotic resistance, and even lethal biosynthetic defects to be phenotypically complemented, thus contributing to the fitness of the community. Motility facilitates cell contact and alignment, which are important in OME and C-signal transmission. All these multicellular-related functions are orchestrated by a complex and interacting gene regulatory network, and although myxobacteriologists have answered many questions, especially related to the regulation of development, there are many interesting issues that remain to be explored. It will be exciting to elucidate the interplay of secondary metabolites in cellular development and in the predatory lifestyle of *M. xanthus*. It remains unknown why myxobacteria have expanded their genomes, why they have such an extraordinary kinome and what roles the large number of signal-transduction systems play in multicellularity. It will probably be possible to answer these and many other interesting questions by using new molecular and microscopy techniques, massive sequencing, transcriptomics, and comparative technologies.

## Author Contributions

All authors listed, have made substantial, direct and intellectual contribution to the work, and approved it for publication.

## Conflict of Interest Statement

The authors declare that the research was conducted in the absence of any commercial or financial relationships that could be construed as a potential conflict of interest.
