# Supplementary material for: Myxobacteria: Moving, Killing, Feeding, and Surviving Together
Source: Front Microbiol. 2016 May 26;7:781. doi: 10.3389/fmicb.2016.00781 (PMC4880591; doi:10.3389/fmicb.2016.00781)
Supplement: Supplementary file 1 [file Table_1.DOCX]

**Table S1**. Genome size and genes encoding proteins involved in signal transduction in the myxobacteria

| **Species** | **Genome size^1^** | **Genes^1^** | **Proteins^1^** | **OC^2^**  **(/Mb)** | **STPK^2^ (/Mb)** | **TCS^3^**  **(/Mb)** | **ECF^2^ (/Mb)** | **Fruiting bodies** | **Reference** |
| --- | --- | --- | --- | --- | --- | --- | --- | --- | --- |
| **Family *Myxococcaceae*^7^ (suborder *Cystobacterineae*)** | | | | | | | | | |
| *Corallococcus coralloides* DSM 2259 | 10.1 | 8054 | 7840 | 412 (40.8) | 106**^4^** (10.5) | 306 (7.5) | 44 (1.1) | YES | Huntley et al., 2012 |
| *Myxococcus fulvus* HW-1 | 9.0 | 7202 | 6912 | 327 (36.3) | 106**^4^** (11.8) | 288 (7.9) | 36 (1.0) | YES | Li et al., 2011 |
| *Myxococcus stipitatus* DSM 14675 | 10.4 | 8053 | 7842 | 420 (40.4) | 113**^4^** (10.9) | 285**^2^** (7.1) | 57 (1.4) | YES | Huntley et al., 2013 |
| *Myxococcus xanthus* DK 1622 | 9.1 | 7248 | 7146 | 315 (34.6) | 100 (11.0) | 282 (8.1) | 41 (1.2) | YES | Goldman et al., 2006 |
| **Family *Cystobacteraceae*^8^ (suborder *Cystobacterineae*)** | | | | | | | | | |
| *Stigmatella aurantiaca* DW4/3-1 | 10.3 | 8213 | 8112 | 488 (47.4) | 209**^4^** (20.3) | 338 (7.1) | 32 (0.7) | YES | Huntley et al., 2011 |
| *Anaeromyxobacter* sp. Fw109-5 | 5.3 | 4551 | 4484 | 152 (28.7) | 20**^4^** (3.8) | 207 (7.2) | 20 (0.7) | NO | Hwang et al., 2015 |
| *Anaeromyxobacter dehalogenans* 2CP-C | 5.1 | 4501 | 4411 | 159 (31.2) | 18**^4^** (3.5) | 188 (6) | 18 (0.6) | NO | Sanford et al., 2002; Thomas et al., 2008 |
| **Family *Polyangiaceae*^9^ (suborder *Sorangiineae*)** | | | | | | | | | |
| *Sorangium cellulosum* So ce56 | 13.0 | 9524 | 9326 | 633 (48.7) | 317**^4^** (24.4) | 273 (5.6) | 86 (1.8) | YES | Schneiker et al., 2007 |
| *Sorangium cellulosum* So0157 | 14.8 | 10808 | 10515 | 613 (41.4) | 508**^5^** (34.3) | 284**^2^** (6.9) | 95 (2.3) | YES | Han et al., 2013 |
| **Family *Haliangiceae*^10^ (suborder *Nannocystineae*)** | | | | | | | | | |
| *Haliangium ochraceum* DSM 14365 | 9.4 | 6823 | 6636 | 441 (46.9) | 229**^4^** (24.4) | 191 (4.1) | 39 (0.8) | YES | Ivanova et al., 2010 |

Data were obtained from: 1, NCBI data base (<http://www.ncbi.nlm.nih.gov/genome/browse/>); 2, MiST 2.2 (<http://mistdb.com/>); 3, Whitworth, 2015; 4, Muñoz et al., 2012; 5, Han et al., 2013; 6, Pérez et al., 2008; 7, Garcia and Müller, 2014b; 8, Knupp Dos Santos, 2014; 9, Garcia and Müller, 2014c; 10, Garcia and Müller, 2014a.

**References**

Gardner, A., and West, S. A. (2006). Demography, altruism, and the benefits of budding. *J. Evol. Biol.* 19, 1707-1716. doi: 0.1111/j.1420-9101.2006.01104.x

Garcia, R., and Müller, R. (2014a). "The family *Haliangiaceae,*" in *The Prokaryotes-*Deltaproteobacteria *and* Epsilonproteobacteria, eds. E. **Rosenberg,** E. F**. DeLong**, S. **Lory**, E. **Stackebrandt**, and F. **Thompson** (Berlin, Heidelberg: Springer-Verlag), 173-181.

Garcia, R., and Müller, R. (2014b). "The family *Myxococcaceae,*" in *The Prokaryotes-*Deltaproteobacteria *and* Epsilonproteobacteria, eds. E. **Rosenberg,** E. F**. DeLong**, S. **Lory,** E. **Stackebrandt,** and F. **Thompson** (Berlin, Heidelberg: Springer-Verlag), 191-212.

Garcia, R., and Müller, R. (2014c). "The family *Polyangiaceae*," in *The Prokaryotes-*Deltaproteobacteria *and* Epsilonproteobacteria*,* eds. E. **Rosenberg,** E. F**. DeLong**, S. **Lory**, E. **Stackebrandt,** and F. **Thompson** (Berlin, Heidelberg: Springer-Verlag), 247-279.

Goldman, B. S., Nierman, W. C., Kaiser, D., Slater, S. C., Durkin, A. S., Eisen, J. A., et al. (2006). Evolution of sensory complexity recorded in a myxobacterial genome. *Proc. Natl. Acad. Sci. U.S.A.* 103, 15200-15205. doi: 10.1073/pnas.0607335103

Han, K., Li, Z. F., Peng, R., Zhu, L. P., Zhou, T., Wang, L. G., et al. (2013). Extraordinary expansion of a *Sorangium cellulosum* genome from an alkaline milieu. *Sci. Rep.* 3: 2101. doi: 10.1038/srep02101

Huntley, S., Hamann, N., Wegener-Feldbrügge, S.,Treuner-Lange, A., Kube, M., Reinhardt, R., et al. (2011). Comparative genomic analysis of fruiting body formation in Myxococcales. *Mol. Biol. Evol.* 28, 1083-1097. doi: 10.1093/molbev/msq292

Huntley, S., Kneip, S., Treuner-Lange, A., and Søgaard-Andersen, L. (2013a). Complete genome sequence of *Myxococcus xanthus* strain DSM 14675 a fruiting myxobacterium. *Genome Announc.* 2: e00100-e00113. doi:10.1128/genomeA.00100-13

Huntley, S., Zhang, Y., Treuner-Lange, A., Kneip, S., Sensen C. W., and Søgaard-Andersen L. (2012). Complete genome sequence of the fruiting *myxobacterium Corallococcus coralloides DSM 2259. J. Bacteriol.* 194, 3012-3013. doi: 10.1128/JB.00397-12

Hwang, C., Copeland, A., Lucas, S., Lapidus, A., Barry, K., Glavina Del Rio, T., et al. (2015). Complete genome of Anaeromyxobacter sp. Fw109-5, an anaerobic, metal reducing bacterium isolated from a contaminated subsurface environment. *Genome* *Announc.* 3: pii: e01449-14. doi: 10.1128/genomeA.01449-14

Ivanova, N., Daum, C., Lang, E., Abt, B., Kopitz, M., Saunders, E., et al. (2010). Complete genome sequence of *Haliangium ochraceum* type strain (SMP-2). *Stand. Genomic Sci.* 2, 96-106. doi: 10.4056/sigs.69.1277

Knupp dos Santos, D. F. K., Kyaw. C. M., De Campos, T. A., Miller, R. N. G., Noronha, E. F., Bustamante, M. M. et al. (2014). “The family *Cystobacteraceae*,” in *The Prokaryotes-*Deltaproteobacteria *and* Epsilonproteobacteria*,* eds. E. **Rosenberg,** E. F**. DeLong,** S. **Lory**, E. **Stackebrandt**, and F. **Thompson** (Berlin, Heidelberg: Springer-Verlag), 19-40.

Li, Z.F., Li, X., Liu, H., Han, K.,Wu, Z.H., Hu,W., et al. (2011). Genome sequence of the halotolerant marine bacterium *Myxoccocus fulvus* HW-1. *J Bacteriol*. 193, 5015-5016. doi: 10.1128/JB.05516-11

Muñoz-Dorado, J., Higgs, P. I., and Elias-Arnanz, M. (2014). "Abundance and complexity of signalling mechanisms in myxobacteria," in *Myxobacteria: genomics, cellular and molecular biology*, eds. Z. Yang, and P. I. Higgs (Norfolk: Caister Academic Press), 127-149.

Pérez, J., Castañeda-García, A., Jenke-Kodama, H., Müller, R., and Muñoz-Dorado J. (2008). Eukaryotic-like protein kinases in the prokaryotes and the myxobacterial kinome. *Proc. Natl. Acad. Sci. U.S.A.*105, 15950-15955. doi: 10.1073/pnas.080685110

Sanford, R. A., Cole, J. R., and [Tiedje, J. M](http://www.ncbi.nlm.nih.gov/pubmed/?term=Tiedje%20JM%5BAuthor%5D&cauthor=true&cauthor_uid=11823233). (2002). Characterization and description of *Anaeromyxobacter dehalogenans* gen. nov., sp. nov., an aryl-halorespiring facultative anaerobic myxobacterium. *Appl. Environ. Microbiol.* 68, 893-900. doi: 10.1128/AEM.68.2.893-900.2002

Schneiker, S., Perlova, O., Kaiser, O., Gerth, K., Alici, A., Altmeyer, M. O., et al. (2007). Complete genome sequence of the myxobacterium *Sorangium cellulosum*. *Nat. Biotechnol.* 25, 1281-1289. doi:10.1038/nbt1354

Thomas, S. H., Wagner, R. D., Arakaki, A. K., Skolnick, J., Kirby, J. R., Shimkets, L. J., et al. (2008). The mosaic genome of *Anaeromyxobacter dehalogenans* strain 2CP-C suggests an aerobic common ancestor to the delta-proteobacteria. *PLoS One* 3 :e2103. doi: 10.1371/journal.pone.0002103

Whitworth, D. E. (2015). Genome-wide analysis of myxobacterial two-component systems: genome relatedness and evolutionary changes. *BMC Genomics* 16: 780. doi: 10.1186/s12864-015-2018-y
